# Fluoroscopic anterior approach versus ultrasound guided superior hypogastric plexus neurolysis in cancer pelvic pain: a randomized controlled study

**DOI:** 10.1186/s12871-022-01948-3

**Published:** 2022-12-27

**Authors:** Nevert A. Abdelghaffar, Tamer Elmetwally Farahat

**Affiliations:** Department of Anesthesia, Intensive Care and Pain Management, Faculty of Medicine, Mansoura University, PO: 35516, Mansoura, Egypt

**Keywords:** Superior hypogastric plexus, Fluoroscopic anterior approach, Ultrasound, Pelvic pain

## Abstract

**Background:**

Cancer-related pelvic pain can be difficult and debilitating to treat. Superior hypogastric plexus neurolysis (SHPN) is a good choice for adequate pain relief with fewer side effects. The current study compared between fluoroscopic anterior approach and ultrasound guided SHPN in the management of cancer-related pelvic pain.

**Methods:**

Patients were randomly allocated into two equal groups. The ultrasound group (US group) (*n* = 48) received SHPN by an ultrasound-guided anterior approach using 3 ml 5% bupivacaine plus 20 ml 10% phenol, while the fluoroscopy group (*n* = 48) received SHPN by a fluoroscopy-guided anterior approach using 3 ml 5% bupivacaine plus 20 ml 10% phenol.

**Results:**

The time of the procedure was shorter in the fluoroscopic group (21.31 ± 4.79 min) than the US group (24.88 ± 6.02 min) (*P* = 0.002). Patient satisfaction was higher in the fluoroscopy group (5.38 ± 1.482) than the US group (2.98 ± 1.495) (P˂0.001). The need for analgesia using morphine was significantly limited in each group, at 1, 2 and 3 months intervals (P_1_˂0.001, P_2_ ˂0.001 and P_3_ ˂0.001). There were statistically significant differences between both groups regarding fatigue at baseline, drowsiness at 3 months, nausea and vomiting at 1, 2 and 3 months and anorexia at 3 months. Group comparison also revealed statistically significant differences regarding depression at one month, anxiety at 2 and 3 months and insomnia at baseline.

**Conclusion:**

The fluoroscopic anterior approach SHPN was more superior than the US guided SHPN regarding the time of the procedure and patient satisfaction, while both technique were similar regarding the numeric rating scale and the complications during block.

**Trial registration:**

Registered in the ClinicalTrials.gov (Identifier: NCT05299047) at 28/03/2022.

## Background

Cancer-related pelvic pain can be difficult and debilitating to treat. Superior hypogastric plexus neurolysis (SHPN) is a good choice for adequate pain relief with fewer side effects and better quality of life in this sector of patients [1].

The superior hypogastric plexus (SHP) is a sympathetic paravertebral retroperitoneal ganglion which is located in upper part of the sacrum and the lower border of the L5 vertebra. It is the continuation of the lumbar sympathetic ganglia and the celiac plexus, and related to the bifurcation of the aorta and the ureter. The SHP has a sympathetic connection (both afferent and efferent fibers) with the aortic plexus and splanchnic nerves. It innervates the viscera of the pelvis, including the upper vagina, the sigmoid colon down to the anal canal, the urinary bladder and ureters [2].

SHP blockade can be guided either by the fluoroscopy, ultrasonography (US), computed tomography (CT) and magnetic resonance imaging (MRI) through either posterior (lateral, para-median, oblique, trans-discal, or trans-vaginal) or anterior (trans-abdominal) approaches [3].

These different imaging modalities and approaches have been described for SHPN trying to make it easier, safer, more accurate and satisfactory to the patients [4].

From this point, we conducted the current study to evaluate the differences between the fluoroscopic anterior approach SHPN and the US guided SHPN in the management of cancer-related pelvic pain regarding the symptoms burden using the Edmonton Symptom Assessment System (ESAS), the time of the procedure, daily analgesic requirements, complications and patient satisfaction.

## Methods

### Study design and participants

This prospective randomized open-labeled endpoint comparative non inferiority study was carried out in the pain clinics of Mansoura University from February 2022 to August 2022, and included 96 participants. The study was approved by the Institutional research board of Faculty of Medicine - Mansoura University (R.22.01.1598) at 21/02/2022. It was registered in the ClinicalTrials.gov (Identifier: NCT05299047) at 28/03/2022 and carried out in compliance with the Helsinki Declaration. All details of every aspect were explained to every patient participating in this study before they signed an informed written consent.

We included patients with cancer-related pelvic pain, of both genders, aged more than 18 years old. We limited the enrolled patients to those who were cancer staging I and II, severe side effects with the opioid therapy or poor pain control and the numeric rating scale (NRS) of pain equal or more than four that ranged from zero (no pain) to ten (extreme pain), American society of Anesthesiology Physical Status class I and II and the body mass index less than 30.

Diagnostic block was done a day before the procedure by injecting 10 ml 0.25% bupivacaine. It should be positive to be included in this study.

The exclusion criteria were patient rejection, grades 2 and 3 ascites, coagulopathy, local or systemic sepsis, distorted local anatomy, and the history of drug abuse, allergy to the used medications, unstable cardiovascular diseases, respiratory problems and previous neurological or psychiatric disorders.

All patients expressed their pain by using the numerical rating score (NRS) from zero to ten.

### Sample size calculation

Sample size was calculated using Power Analysis and Sample Size software program (PASS) version 2021 using the data published by Abdelghafar et al. (2021) with patient global impression of change (PGIC) score 12 weeks after the procedure as the primary outcome [2]. The null hypothesis was considered as the presence of a difference between both treatment modalities regarding the PGIC score. According to Abdelghafar et al. [2], the PGIC score after 3 months was 3.5 (2-5) in the ultrasound group and 4 (2-5) in the fluoroscopy group. A non-inferiority margin of 10% (0.375) of the mean PGC score in the fluoroscopy group at 12 weeks was set as the target non-inferiority margin between both groups. A sample size of 38 patients in each group was needed to achieve 95% power (the probability of making type II error as rejecting the null hypothesis when it is actually false = β) in the suggested study using one-sided, two-sample unequal-variance t-test with a significance level (the probability of making type I error as rejecting the null hypothesis when it is actually true = α) of 5%. 10 drop-out patients were predictable in each group, so a total of 48 patients were enrolled into each group.

### Statistical analysis

The statistical analysis of data was done by using Statistical Package for Social Science (SPSS) program (version 22). The normality of data distribution was tested by Shapiro-Wilk test for only significant data appeared to be nonparametric. The numerical variables between-group was compared by using unpaired student-t test, if its assumptions were fulfilled. Otherwise, the Mann–Whitney test was used for non-parametric. Mean (±SD) was done for quantitative data. Qualitative data was represented by frequency and proportion using the chi-square test. Any change or difference in groups showing probability (P) less than 0.05 was considered statistically significant at the confidence interval 95%.

### Randomization

**Fig. 1:**
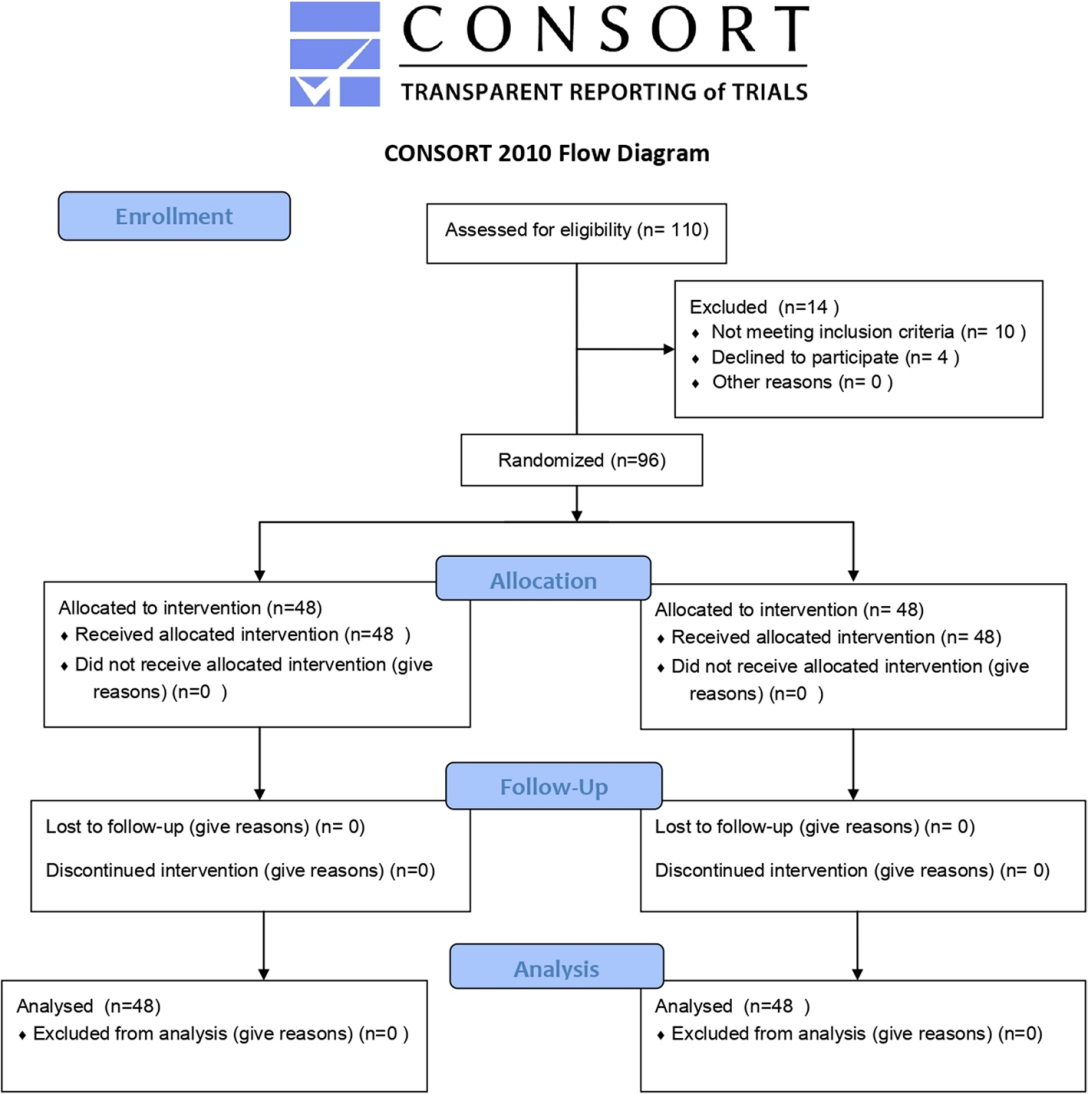
Consort flow chart

At the time of the first visit to the pain clinic, the randomization was done using closed envelopes indicating the group of the assignment by one of our team who did not contribute in patients’ follow-up. He looked at the number inside the envelope and specified group assignments (Fig. 1). Patients were randomly classified into two equal groups:

- US guided group (*n* = 48) received SHPN by the US guided anterior approach using 3 ml 5% bupivacaine plus 20 ml 10% phenol.
- Fluoroscopy guided group (*n* = 48) received SHPN by the fluoroscopy guided anterior approach using 3 ml 5% bupivacaine plus 20 ml 10% phenol.

### Procedures

All procedures were done under complete aseptic conditions. Before the procedure, 18-G intravenous catheter was secured and patients received 1000 ml of the lactated ringer solution to avoid hypotension. The vital parameters of the patients (peripheral oxygen saturation, heart rate and non-invasive blood pressure) were continuously observed during and two hours after the procedure. Intravenous sedation was carried out using 0.1 mg/kg midazolam and 1 µg/kg fentanyl.

### SHPN by the US guided anterior approach [4, 5]

**Fig. 2:**
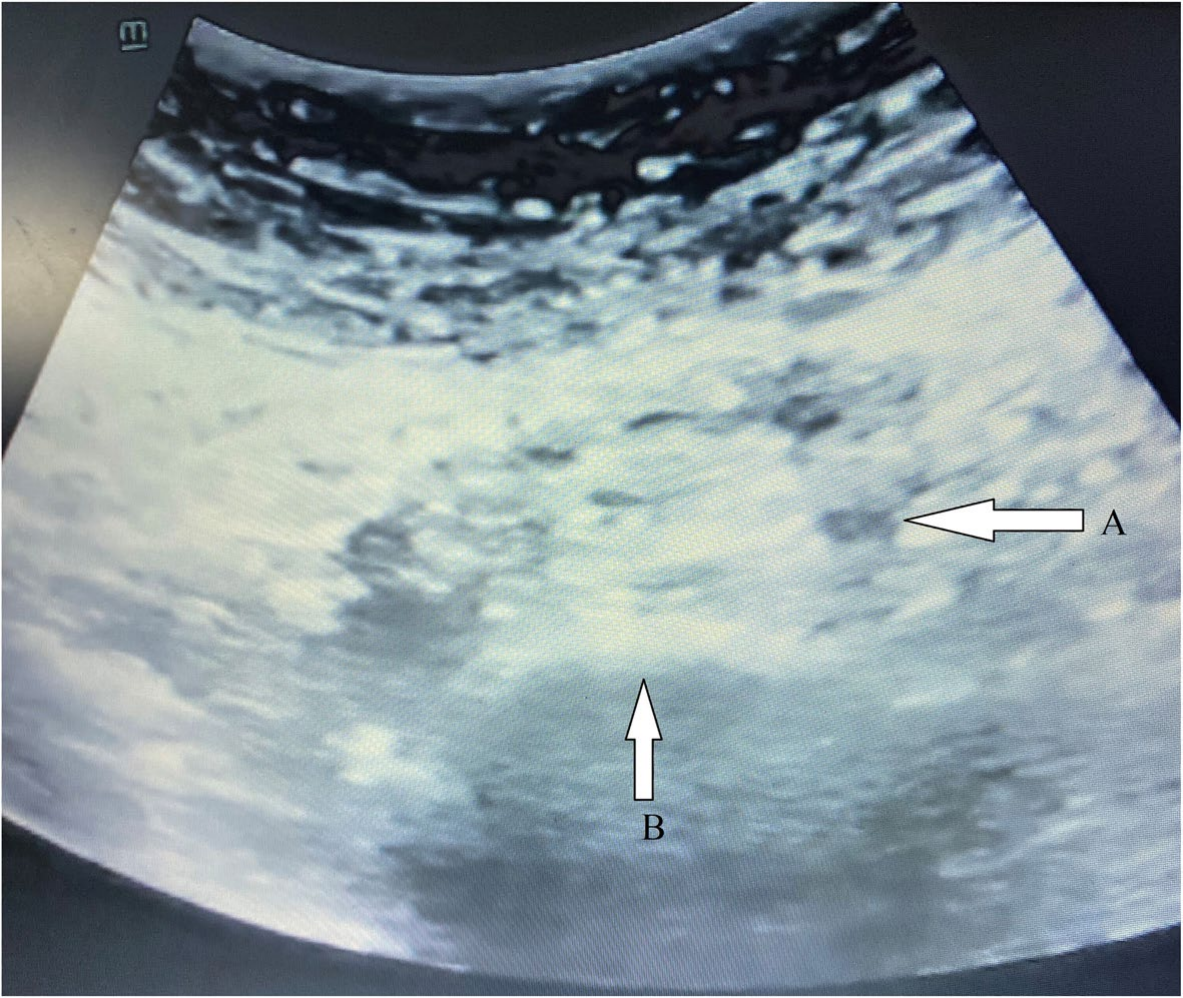
Ultrasound view showed the common iliac vessels (**A**) and the body of the fifth lumber vertebra (**B**)

**Fig. 3:**
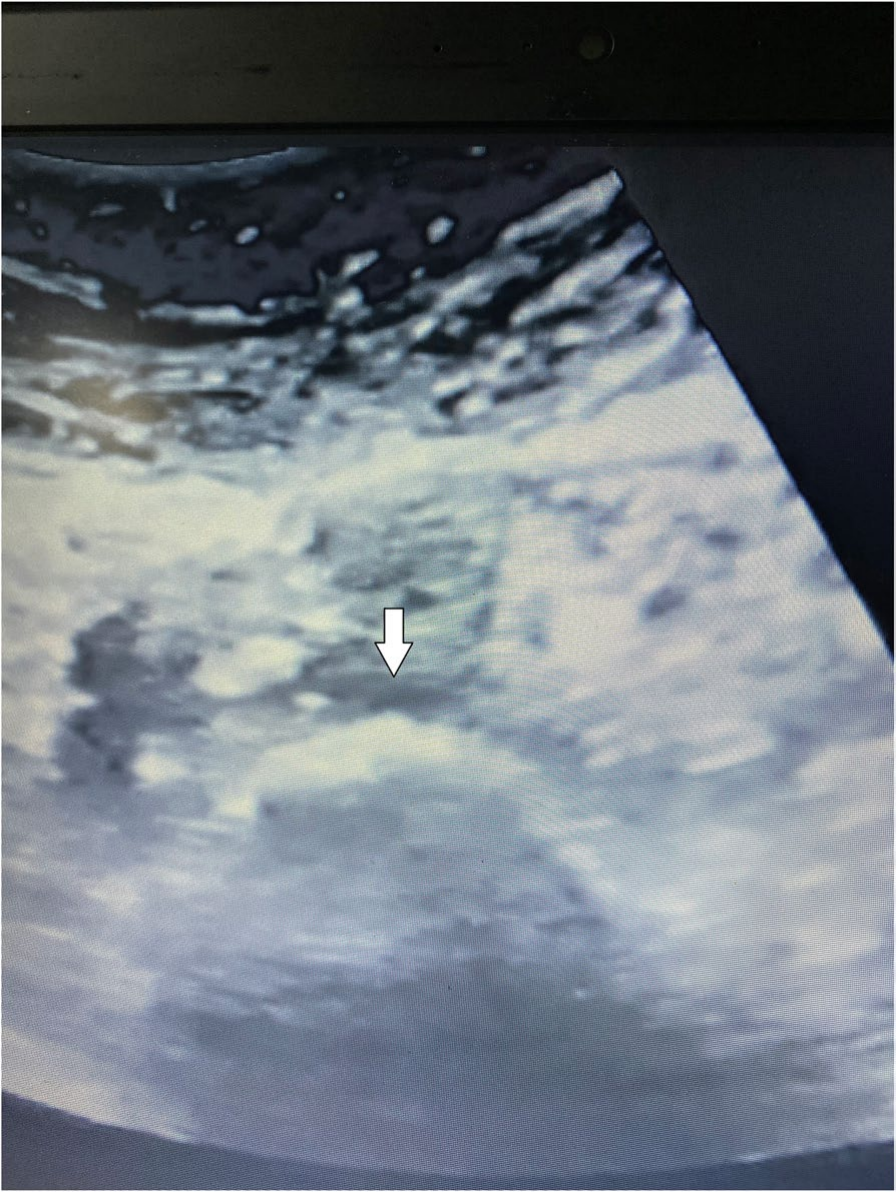
Ultrasound view showed the phenol in front of the body of the fifth lumber vertebra (white arrow)

The night before the procedure, two tablets of bisacodyl and four tablets of activated charcoal were given to clean the bowel of contents and air. The patients stopped eating for eight hours before the procedure. Prior to the procedure, the patients were advised to micturate to empty the urinary bladder. By using the oblique ultrasonography with a 5-2 MHz curved transducer, the dissection of the abdominal aorta into the common iliac arteries was located and the vertebral body of L5 was imaged, at which the bilateral common iliac vessels were seen leaving the midline (Fig. 2). Color Doppler imaging was used to confirm the location of the common iliac vessels. Lignocaine solution 2% (3–4 ml) was injected below the umbilicus to provide local cutaneous and subcutaneous anesthesia. Out-of-plane technique, a 20 cm long, 22-gauge Chiba needle was introduced into the hypogastrium to access the most anterior point of the fifth lumbar vertebral body, so that injected drug spread equally bilateral along the anterior curvature of the fifth lumbar vertebral body. The needle was withdrawn 1 mm after hitting the fifth lumber vertebral body to avoid the periosteal position of the needle tip, then aspiration was applied to avoid that the needle was within a vessel. Then, 3 ml of 0.5% bupivacaine was injected, if there is no variation in heart rate or change in neurological status and 20 ml of 10% phenol was slowly injected for neurolysis (Fig. 3).

### SHPN by the fluoroscopy guided anterior approach technique [6]

**Fig. 4:**
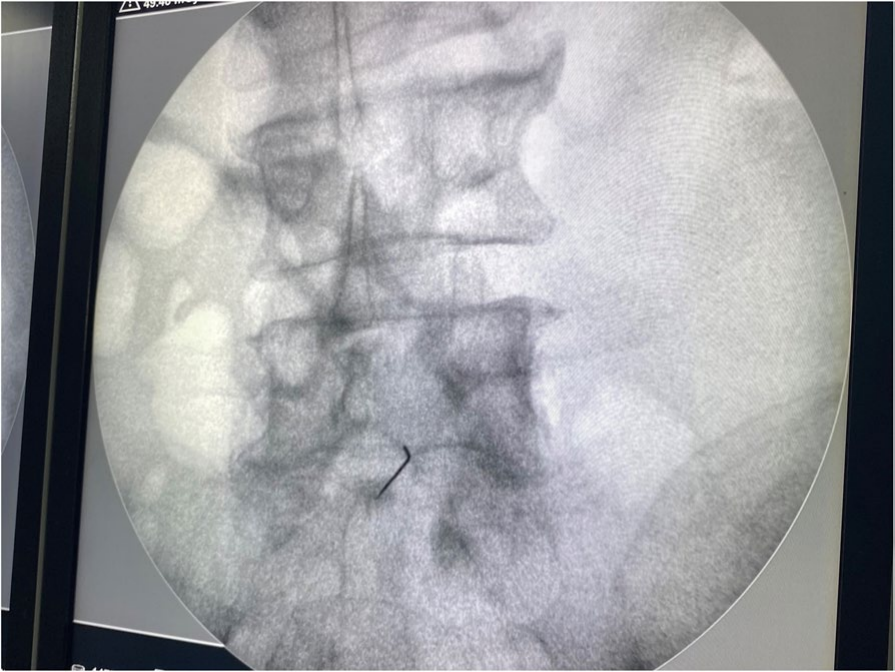
Fluoroscopic anterior view showed the needle at the level of the body of the fifth lumber vertebra

**Fig. 5:**
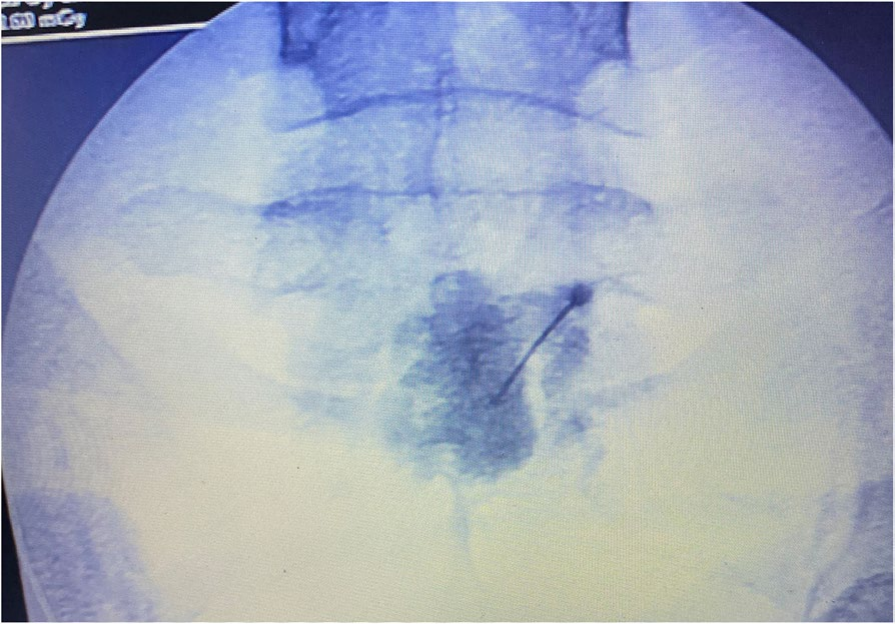
Fluoroscopic anterior view showed the contrast distribution

**Fig. 6:**
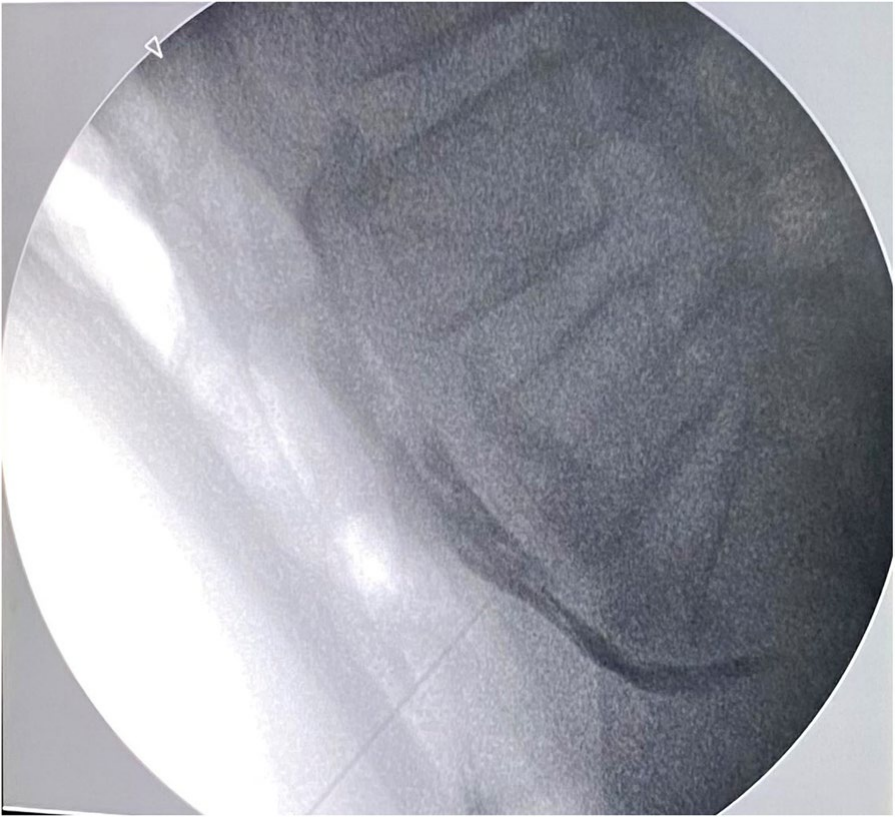
Fluoroscopic lateral view showed the contrast distribution in front of the vertebrae

The patient was positioned in the supine position. The L5-S1 inter-discal space was identified using fluoroscopy. Under the sterile conditions, the entry site of the needle was visualized using a radiopaque object (like a needle) on the skin. Lignocaine solution 2% (3–4 ml) was injected below the umbilicus to provide local cutaneous and subcutaneous anesthesia. Under the ongoing fluoroscopic guidance, a 20 cm long, 22-gauge Chiba needle was advanced to the anterior portion of the fifth vertebral body (Fig. 4). Once the needle reached bony resistance, 2–5 ml contrast (iohexol-omnipaque 300 mg iodine/mL) was gently injected and revealed no vascular opacification with the characteristic triangular blob of the contrast (Fig. 5). Then, lateral view was taken to confirm the crescent contrast which directly spread in front of the vertebral body (Fig. 6).

**Fig. 7:**
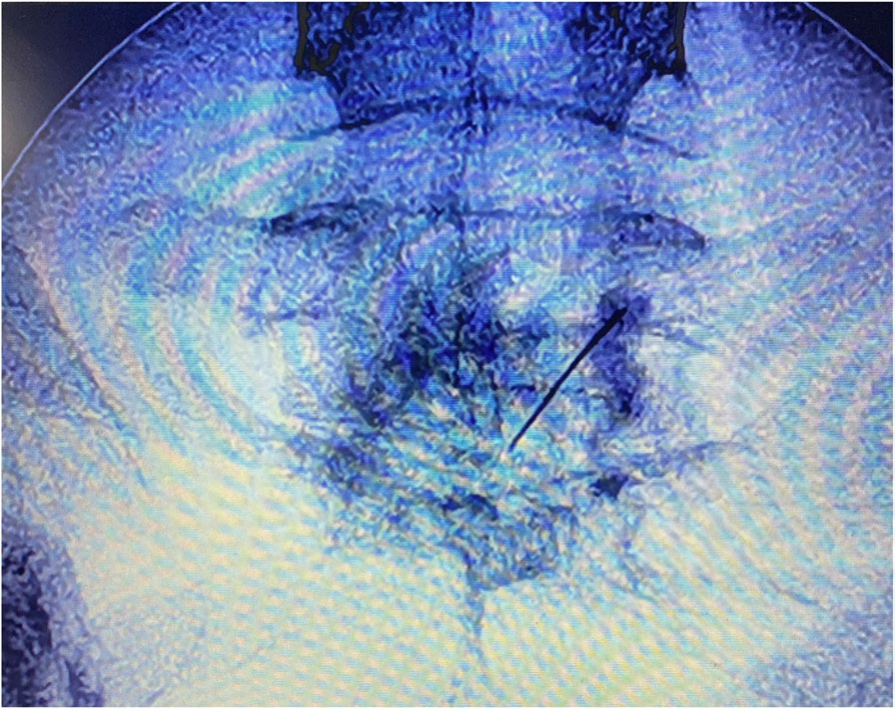
Fluoroscopic anterior view showed the phenol distribution

Before the injection, aspiration was done to avoid any blood. Bupivacaine 0.5% (3 ml) was then injected as a preliminary test to confirm that there was no change in the neurological status or heart beats, and then 20 ml of phenol 10% was slowly injected with intermittent aspiration (Fig. 7). During the injection, a slight forward pressure was applied on the needle in order to avoid withdrawal into other structures.

Any problems or complications associated with the method were noted and managed.

The following parameters were monitored in every patient: (1) The symptom burden was evaluated using the Edmonton Symptom Assessment System (ESAS) before and 1, 2, 3 months after the SHPN. This tool assessed ten symptoms with a range from 0 to 10 including: pain (numerical rating scale NRS), fatigue, drowsiness, anorexia, nausea, feeling of well-being, anxiety, depression, shortness of breath, and insomnia [7]; (2) Time of the procedures; (3) The number of patients who needed analgesia; (4) complications; (5) Patient satisfaction which assessed by using a linear scale range from zero to ten in which 0 was very dissatisfied and 10 was very satisfied.

## Result

Table 1 shows no statistically significant difference between both groups as regards the demographic characteristics, complications and the number of patients who needed analgesia. The time of the procedure was shorter in the fluoroscopy group (21.31 ± 4.79 minutes) than the US group (24.88 ± 6.02 minutes) (*P*=0.002). Patient satisfaction was higher in the fluoroscopy group (5.38 ± 1.482) than the US group (2.98 ± 1.495) (P <0.001). Within-group comparison revealed limited need for analgesia using morphine in each group at 1, 2 and 3 months intervals (*P*_1_<0.001, *P*_2_<0.001 and *P*_3_<0.001). While between-group comparison shows no statistically significant differences at 1, 2, 3 months (*P*= 0.573, 0.601 and 0.82, respectively).

**Table 1:** Demographic data, time of the procedure, patient satisfaction, complications and the number of patients who needed analgesia

	US group (*n* = 48)	Fluoroscopy group (*n* = 48)	*P*
Age	38.35 ± 10.977	40.33 ± 10.360	0.366
Gender			
Male	16 (33.3%)	17 (35.4%)	0.83
Female	32 (66.7%)	31 (64.6%)	
Time of the procedure (minutes)	24.88 ± 6.02	21.31 ± 4.79	0.002^*^
Patient satisfaction	2.98 ± 1.495	5.38 ± 1.482	< 0.001^*^
Complications			
Hypotension	8 (16.7%)	7 (14.6%)	0.779
Blood aspirate	0	2 (4.2%)	0.153
Bradycardia	1 (2.1%)	0	0.315
Visceral injury	0 (0.0%)	0	1
The number of patients who needed analgesia (morphine 60 mg/day)			
Total number	48 (100%)	48 (100%)	1
1 month	7 (14.6%)	5 (10.4%)	0.573
P_1_	< 0.001^*^	< 0.001^*^	
2 months	10 (20.8%)	8 (16.7%)	0.601
P_2_	< 0.001^*^	< 0.001^*^	
3 months	13 (27.1%)	14 (29.2%)	0.82
P_3_	< 0.001^*^	< 0.001^*^	

Data is expressed as mean and standard deviation or as percentage and frequency. P_1_ represents the number of patients at 1 month vs. the total number; P2 represents the number of patients at 2 month vs. the total number; P_3_ represents the number of patients at 3 month vs. the total number

Table 2 shows no statistically significant difference between both groups as regards NRS, fatigue at 1, 2 and 3 months, drowsiness at 1 and 2 months, and anorexia at 1 and 2 months. Meanwhile, there were statistically significant differences between both groups regarding the fatigue at baseline (*P*=0.015), drowsiness at 3 months (*P*=0.027), nausea and vomiting at 1, 2 and 3 months (*P*=0.015, 0.002, 0.001, respectively) and anorexia at 3 months (*P*=0.005). Within each group, statistically significant difference were reported regarding the NRS, fatigue, drowsiness at 2 and 3 months, nausea and vomiting and anorexia, when compared to the baseline values.

**Table 2:** NRS, fatigue, drowsiness, nausea and vomiting and lack of appetite in both groups at interval 1, 2, 3 months and between each interval to the baseline in each group

	US group (*n* = 48)	Fluoroscopy group (*n* = 48)	95% CI	*P*
NRS				
Baseline	7.60 ± 1.026	7.40 ± 1.125	-0.2, 0.6	0.346
1 month	2.56 ± 1.398	2.40 ± 1.469	-0.4, 0.7	0.57
P_1_	< 0.001^*^	< 0.001^*^		
2 months	2.98 ± 1.48	2.81 ± 1.379	-0.4, 0.7	0.569
P_2_	< 0.001^*^	< 0.001^*^		
3 months	3.52 ± 1.473	3.40 ± 1.216	-0.4, 0.7	0.651
P_3_	< 0.001^*^	< 0.001^*^		
Fatigue				
Baseline	7.42 ± 1.028	6.75 ± 1.564	0.1, 1.2	0.015^*^
1 month	4.73 ± 1.233	4.77 ± 1.171	-0.5, 0.4	0.866
P_1_	< 0.001^*^	< 0.001^*^		
2 months	5.15 ± 1.130	4.79 ± 1.184	-0.1, 0.8	0.137
P_2_	< 0.001^*^	< 0.001^*^		
3 months	5.38 ± 1.265	4.88 ± 1.196	0.0, 1	0.05
P_3_	< 0.001^*^	< 0.001^*^		
Drowsiness				
Baseline	2.63 ± 1.378	2.52 ± 1.544	-0.5, 0.7	0.728
1 month	2.50 ± 1.111	2.25 ± 1.376	-0.3, 0.8	0.33
P_1_	0.598	0.298		
2 months	2.19 ± 0.960	2.52 ± 1.238	-0.8, 0.1	0.144
P_2_	0.026^*^	1		
3 months	2.08 ± 1.145	2.65 ± 1.296	-1.1, -0.1	0.027^*^
P_3_	0.046^*^	0.0676		
Nausea and vomiting				
Baseline	6.08 ± 1.661	6.29 ± 1.762	-0.9, 0.5	0.553
1 month	2.52 ± 1.271	3.25 ± 1.605	-1.3, -0.1	0.015^*^
P_1_	< 0.001^*^	< 0.001^*^		
2 months	2.25 ± 1.229	3.10 ± 1.387	-1.4, -0.3	0.002^*^
P_2_	< 0.001	< 0.001^*^		
3 months	1.96 ± 1.237	2.94 ± 1.616	-1.6, -0.4	0.001^*^
P_3_	< 0.001^*^	< 0.001^*^		
Anorexia				
Baseline	6.25 ± 1.509	6.46 ± 1.774	-0.9, 0.5	0.537
1 month	2.90 ± 1.207	3.13 ± 1.734	-0.8, 0.4	0.454
P_1_	< 0.001^*^	< 0.001^*^		
2 months	3.10 ± 1.477	2.73 ± 1.526	-0.2, 1	0.224
P_2_	< 0.001^*^	< 0.001^*^		
3 months	3.67 ± 1.693	2.77 ± 1.356	0.3, 1.5	0.005^*^
P_3_	< 0.001^*^	< 0.001^*^		

Data is expressed as mean and standard deviation. 95% CI: 95% confidence interval of the mean difference between both groups. P_1_ represents the values at 1 month vs. baseline; P_2_ represents the values at 2 month vs. baseline; P_3_ represents the values at 3 month vs. baseline

Table 3 shows no statistically significant differences between both groups regarding the depression at 2 and 3 months, anxiety at one month, shortness of breath, feeling of well-being and insomnia. Meanwhile, there were statistically significant differences between both groups regarding the depression at one month (*P*=0.029), anxiety at 2 and 3 months (*P*=0.005 and 0.008, respectively) and insomnia at the baseline (*P*=0.024). Within each group, there were statistically significant differences regarding the depression in 1, 2 and 3 months, anxiety, feeling of well-being and insomnia, when compared to the baseline values (*P*_*1*_<0.001, *P*_*2*_ <0.001 and *P*_*3*_ <0.001).

**Table 3:** Depression, anxiety, shortness of breath, feeling of well-being and insomnia in both groups at interval 1, 2, 3 months and between each interval to the baseline in each group

	US group (*n* = 48)	Fluoroscopy group (*n* = 48)	95% CI	*P*
Depression				
Baseline	7.17 ± 1.277	6.85 ± 1.368	-0.2, 0.8	0.25
1 month	3.98 ± 1.407	4.63 ± 1.453	-1.2, -0.1	0.029^*^
P_1_	< 0.001^*^	< 0.001^*^		
2 months	3.65 ± 1.537	3.69 ± 1.24	-0.6, 0.5	0.884
P_2_	< 0.001^*^	< 0.001^*^		
3 months	4.21 ± 1.75	3.71 ± 1.148	-0.1, 1.1	0.101
P_3_	< 0.001^*^	< 0.001^*^		
Anxiety				
Baseline	5.85 ± 1.856	6.31 ± 1.475	-1.1, 0.2	0.184
1 month	3.94 ± 1.156	3.83 ± 1.389	-0.4, 0.6	0.691
P_1_	< 0.001^*^	< 0.001^*^		
2 months	4.48 ± 1.111	3.69 ± 1.546	0.2, 1.3	0.005^*^
P_2_	< 0.001^*^	< 0.001^*^		
3 months	4.38 ± 1.393	3.56 ± 1.556	0.2, 1.4	0.008^*^
P_3_	< 0.001^*^	< 0.001^*^		
shortness of breath				
Baseline	3.73 ± 1.594	3.38 ± 1.248	-0.2, 0.9	0.229
1 month	3.35 ± 1.644	3.35 ± 1.329	-0.6, 0.6	1
P_1_	0.095	0.941		
2 months	3.54 ± 1.663	3.54 ± 1.184	-0.6, 0.6	1
P_2_	0.381	0.514		
3 months	3.60 ± 1.865	3.17 ± 1.226	-0.2, 1.1	0.178
P_3_	0.658	0.474		
Feeling of well-being				
Baseline	6.08 ± 1.471	6.42 ± 1.456	-0.9, 0.3	0.267
1 month	4.06 ± 1.63	3.56 ± 1.486	-0.1, 1.1	0.12
P_1_	< 0.001^*^	< 0.001^*^		
2 months	3.96 ± 1.701	3.60 ± 1.608	-0.3, 1.0	0.297
P_2_	< 0.001^*^	< 0.001^*^		
3 months	3.90 ± 1.893	3.69 ± 1.291	-0.4, 0.9	0.53
P_3_	< 0.001^*^	< 0.001^*^		
Insomnia				
Baseline	6.52 ± 1.220	7.23 ± 1.765	-1.3, -0.1	0.024^*^
1 month	3.15 ± 1.502	3.40 ± 1.364	-0.8, 0.3	0.395
P_1_	< 0.001^*^	< 0.001^*^		
2 months	3.04 ± 1.166	3.31 ± 1.417	-0.8, 0.3	0.309
P_2_	< 0.001^*^	< 0.001^*^		
3 months	3.5 ± 1.611	3.60 ± 1.512	-0.7, 0.5	0.745
P_3_	< 0.001^*^	< 0.001^*^		

Data is expressed as mean and standard deviation. 95% CI: 95% confidence interval of the mean difference between both groups. P_1_ represents the values at 1 month vs. baseline; P_2_ represents the values at 2 month vs. baseline; P_3_ represents the values at 3 month vs. baseline

## Discussion

Chronic pelvic cancer pain is a major distressing problem. There are several lines of treatment such as surgical, pharmacological and neurolytic blocks [8]. Although opioids stay the cornerstone of cancer pain management, they produce many harmful side effects with negative impacts on the patient’s life [9, 10].

Blockade of SHP has been proven to be successful in relieving cancer pelvic pain [11]. The classic fluoroscopy-guided posterior approach for the SHPN is the standard technique. However, the prone position for patients suffering from abdominal or pelvic cancer pain is very painful and annoying. Moreover, the airway is not secured if the patient is sedated and the immediate airway management is difficult in this position [12].

Mishra et al reported that cancer patient is having difficulty in lying prone [13]. As well Kamel et al compared between fluoroscopic posterior versus US-guided anterior approach for SHPB and reported that the anterior approach reduced the discomfort because it prevents passing the needles through the back muscles and avoids L5 nerve root injury and it may be the only appropriate technique in patients with progressive spondylodegenerative changes of the backbone [3].

On the other hand, the anterior approach carries the risk of infections and injuries of the common iliac vessels, the bowel and the urinary bladder. Bhantagar et al stated that these hazards can be minimized by using the proper bladder and bowel preparation, trendelenburg position, smaller size Chiba needle, and using US with Doppler [14]. Although Mishra et al reported that ultrasound cannot absolutely exclude intravascular drugs injection [15].

Despite the safety and benefits of ultrasound in showing vessels and organs, it requires an expert personnel to perform the block [3]. While fluoroscopy anatomy is more easily understood [16]. Also fluoroscopy is important to verify the needle position and the spread of the contrast [4]. So in our study, we compared the fluoroscopy in the anterior approach versus ultrasound SHB.

Both groups showed improvement in the pain score with no significant differences. Unlike the previous study, which reported statistically significant differences regarding the NRS between both groups, being better in the ultrasound group than the fluoroscopic posterior approach group at the 1^st^, 4^th^, and 8^th^ week (*P*<0.01) [2].

In the current study, the time of the procedure was shorter in the fluoroscopy group in the supine position than the US group. This finding is in contrast with Abdelghafar et al who reported longer time of the procedure in the fluoroscopy group in prone position (30±6.4) than the ultrasound group in supine position (17.33±3.166) [2].

Patients with cancer cannot tolerate prolonged procedures, so the time of the procedure is an important factor in patient satisfaction. This was proven in the current study by finding that patients in fluoroscopy group with the shorter procedure time were more satisfied than the US group.

In the current study, we did not report serious complication such as bowel perforation, mostly due to the proper insertion technique and the accurate needle sizes. Regarding the vessel puncture, we only reported two patients with tinged blood aspiration. We injected dye and took several fluoroscopic photos to exclude dye extension into any vessels. We also injected 3 ml of 0.5% bupivacaine as a preliminary test to confirm that there was no change in neurological status or heart beats.

No statistically significant difference existed between both groups regarding the complication. The advantage of ultrasonography in avoiding vascular puncture could be overcome by the fluoroscopic anterior approach technique by aspiration, injection of contrast and the entrance of the needle in the middle L5-S1 while the bifurcation of abdominal aorta in cancer pelvic patient existed above the L4-L5 intervertebral spaces in 81.3% of these patients [17].

Approximately 30% to 50% of the cancer patients suffer from insomnia which is common among those patients and is associated with psychological disorders such as anxiety or depression. The relationship between cancer pain, fatigue, insomnia, depression and anxiety are complex, so warranting treatment focus not only on the pain relief to improve quality of life but also to improve other related symptoms [18]. After SHPN, we found a statistically significant reduction in these symptoms in both groups, compared to the baseline.

Opioids have many side effects which can limit their use [19]. SHPN could manage and control pain making it possible to dose opioids to adequate analgesia with tolerable side effects. The need for morphine was significantly reduced in both groups, thus reduced the feeling of nausea and anorexia [20].

However the difference in the aforementioned symptoms between both groups could be attributed to other variables such as chemotherapy. Rao and Faso reported that nausea and vomiting are serious complication of chemotherapy [21]. Symptoms of depression and anxiety in cancer patients were also independently associated with chemotherapy-induced nausea [22]. So we need further large scale studies with extended periods of follow-up to verify our findings.

## Limitation

The current study was a single center study with a relatively short follow-up period and did not include all stages of cancer pelvis.

## Conclusion

Anterior approach fluoroscopy SHPN is more superior than the US guided SHPN regarding the time of the procedure and patient satisfaction while both technique were similar regarding NRS and complications during block. We recommend larger extended studies including more advanced cancer stages to generalize our findings.

## Data Availability

The datasets used and/or analyzed during the current study are available from the corresponding author on reasonable request.

## Abbreviations

SHPN: Superior hypogastric plexus neurolysis
US: Ultrasound
CT: Computed tomography
MRI: Magnetic resonance imaging
NRS: Numeric rating scale
PASS: Power Analysis and Sample Size software program
PGIC: Patient global impression of change
SPSS: Statistical Package for Social Science
ESAS: Edmonton Symptom Assessment System
